# Special Issue “Advances in Targeted Cancer Therapy and Mechanisms of Resistance—2nd Edition”

**DOI:** 10.3390/ijms26157173

**Published:** 2025-07-25

**Authors:** Patrick Ming-Kuen Tang, Yan-Fang Xian, Dongmei Zhang

**Affiliations:** 1 Department of Anatomical and Cellular Pathology, State Key Laboratory of Translational Oncology, Peter Hung Pain Research Institute, Li Ka Shing Institute of Health Sciences, Prince of Wales Hospital, The Chinese University of Hong Kong, Hong Kong; 2 School of Chinese Medicine, Faculty of Medicine, The Chinese University of Hong Kong, Hong Kong; lisaxian@cuhk.edu.hk; 3 College of Pharmacy, Jinan University, Guangzhou 510632, China; dmzhang701@jnu.edu.cn

Conventional therapy commonly leads to drug resistance, off-targeted effects, and even lethal complications in cancer patients [1]. With the advent of sequencing technologies with single-cell resolution [2], precision therapeutic targets and molecular mechanisms for drug resistance and complications can be identified in a treatment- and disease-type-specific manner, providing a fundamental improvement in cancer therapy.

Indeed, drug resistance is a major burden in clinics, including both secondary and primary resistance in cancer patients who have received conventional therapy, targeted therapy, and the latest immunotherapy [3]. The molecular mechanism in solid tumors that contributes to drug resistance is important to resolving this clinical burden. Better understanding cancer immunodynamics under conventional therapy can identify clues to further potentiate the efficacy and safety of cancer therapy in clinics [4]. For example, using advanced single-cell bioinformatics, a number of interesting phenomena have been uncovered from clinical samples with advanced single-cell bioinformatics, including the macrophage-to-neuron-like cell transition (MNT) [5,6], the macrophage–myofibroblast transition (MMT) [7,8,9], neutrophil polarization [10,11,12], etc.

In this Special Issue in partnership with the *International Journal of Molecular Sciences*, we are pleased to share seven original articles and two review papers related to the latest findings from cancer therapy, covering (1) drug resistance and (2) novel therapeutics. These high-quality papers cover multiple disciplines, embracing laboratory experiments and clinical observations, and they provide valuable insights into how we can overcome resistance and design novel targeted therapies.

## 1. Drug Resistance

Better understanding cancer immunodynamics under treatment stress would allow us to identify safe and effective strategies to overcome drug resistance in clinics. Bera et al. found BRAF inhibitor resistance in thyroid cancers due to the reduction in Annexin 7 and proposed a p21-dependent strategy for overcoming drug resistance (Contribution 1). Akhtar et al. demonstrated that CtBP1 conferred paclitaxel resistance to esophageal squamous cell carcinoma, where knocking out of CtBP1 not only sensitized them but also markedly inhibited their progression (Contribution 2). Long non-coding RNA is an emerging therapeutic target for inflammatory diseases including cancer [13,14]. Gonzalez-Woge et al. discovered that lncRNA GATA3-AS1 is positively associated with neoadjuvant chemotherapy resistance in luminal B breast cancer patients (Contribution 3). Urushihara et al. highlighted the curial role of the tumor microenvironment in drug resistance. Their findings suggested that AMPK and FOXO3a in a nutrient-deprived microenvironment can initiate radiotherapy resistance, and genetically targeting them can enhance the anticancer effect of radiotherapy via promoting cancer apoptosis under nutrient starvation (Contribution 4). In addition, Mouhssine et al. summarized and discussed how BTK inhibitor resistance occurs in B cell malignancies (Contribution 5). They provide insight to overcome resistance by using the next-generation BTK inhibitors with non-covalent or proteolysis-targeting chimeric features in clinics.

## 2. Novel Therapeutics

A better understanding of drug resistance mechanisms at the molecular level with advanced technology allows for the development of targeted cancer therapy with better efficiency, specificity, and safety [15,16]. With a multi-omics approach, Stocchero et al. demonstrated that LIN28B governed neuroblastoma cell metabolism and contributed to adverse treatment outcomes, suggesting LIN28B as a potential therapeutic target for neuroblastoma (Contribution 6). Zhou et al. established a prediction model by combining RNA-seq and clinical data. They revealed a pathogenic role of CHI3L1 in poor clinical outcomes of glioma, possibly through the modulation of oxidative stress-related genes in cancer cells (Contribution 7). Interestingly, Bashraheel et al. designed tumor-selective superantigen-based peptides, representing precision cancer immunotherapy which can target EGFR-positive cancer cells with better specificity and safety (Contribution 8). Moreover, Sellner et al. discussed how genetic and epigenetic characteristics affect pancreatic metastases in renal cell carcinoma based on a seed and soil hypothesis to explain this specific organotropism (Contribution 9).

## 3. Conclusions

This Special Issue showcases how we can improve targeted therapy and overcome drug resistance in cancer by revisiting clinical data with a multi-omics approach [17,18] and laboratory experimental validation [19,20]. These peer-reviewed papers offer translational findings with clinical relevance to further advance cancer therapy, hopefully leading to safe and effective therapeutic strategies for cancer patients in clinics in the future.
